# Color Occlusion Face Recognition Method Based on Quaternion Non-Convex Sparse Constraint Mechanism

**DOI:** 10.3390/s22145284

**Published:** 2022-07-15

**Authors:** Chenglin Wen, Yiting Qiu

**Affiliations:** 1 School of Automation, Guangdong University of Petrochemical Technology, Maoming 525000, China; 2 School of Automation, Hangzhou Dianzi University, Hangzhou 310018, China; qiu_yt@hdu.edu.cn

**Keywords:** occluded color face, PCANet, *Lp* non-convex sparse, coordinate descent, fixed point iterative

## Abstract

As the acquisition and application of color images become more and more extensive, color face recognition technology has also been vigorously developed, especially the recognition methods based on convolutional neural network, which have excellent performance. However, with the increasing depth and complexity of network models, the number of calculated parameters also increases, which means the training of most high-performance models depends on large-scale samples and expensive equipment. Therefore, the key to the current research is to realize a lightweight model while ensuring the recognition accuracy. At present, PCANet, a typical lightweight framework for deep learning, has achieved good results in most of the image recognition tasks, but its recognition accuracy for color face images, especially under occlusion, still needs to be improved. Therefore, a color occlusion face recognition method based on quaternion non-convex sparse constraint mechanism is proposed in this paper. Firstly, a quaternion non-convex sparse principal component analysis network model was constructed based on *Lp* regularization of strong sparsity. Secondly, the fixed point iteration method and coordinate descent method were established to solve the non-convex optimization problem. Finally, the occlusion recognition performance of the proposed method was verified on Georgia Tech, Color FERET, AR, and LFW-A Color face datasets.

## 1. Introduction

As one of the important biometric recognition technologies, face recognition technology has been more and more widely used in social security, video surveillance, identity verification, mobile payment, and other fields. In recent years, research on face recognition technology have made a series of important achievements [1], especially the deep learning method based on large label samples, which has achieved very high accuracy.

The main drawback of deep learning methods is that they rely on large datasets to train models, and those datasets need to contain enough variation to generalize to previously unseen samples. However, in many real-world use cases, most datasets are not on the scale that deep learning uses, and even small-scale data collection can be very expensive or sometimes nearly impossible [2]. If the deep learning model is trained with small samples, when the model starts to use irrelevant features for prediction, it leads to the over-fitting problem, which greatly limits its classification recognition performance. As the number of network layers increases, the model structure becomes more complex, which greatly increases the amount of computation. This makes most of the advanced deep learning algorithms need to be trained and deployed on expensive high-performance graphics cards, which largely hinders the practical application and development of deep learning.

Therefore, in order to solve the problems of the above deep learning methods, the principal component analysis network (PCANet) was proposed [3]. PCANet is a lightweight deep learning algorithm combining PCA and CNN. It has a simple structure and can achieve good results in most image classification tasks with two-layer convolutional networks. Compared with CNN, it does not need a large number of training samples and the support of highly configured hardware, and the parameters and calculations are also very small while the recognition effect is guaranteed. Moreover, compared with traditional machine learning methods, PCANet has a stronger feature extraction ability. 

At the same time, with the increasing maturity of multimedia equipment and technology, daily visual information has more abundant forms of expression, especially the acquisition and application of color image is more and more extensive. A color face image can obtain more sufficient color information, such as skin color, hair color, etc. Color image has a natural recognition advantage compared with gray image, and color face recognition technology has also attracted the attention of scholars [4]. The famous feature face method proposed by Turk is mainly to extract feature space after converting a color face image to a gray image [5]. After grayscale processing, the dimension of the image matrix decreases and the operation speed increases, but this causes the loss of image color information, and the recognition effect is not good because of not making full use of the characteristics of color. Torres pointed out that color information in color images is composed of RGB in different proportions, which is of great significance for face recognition, and proposed to extend the RGB three-color channels to the traditional PCA method for color face recognition [6]. At the same time, most of the existing convolutional neural networks usually regard RGB color channels as three independent feature channels, and use three convolution kernels to convolve and add the three channels respectively in the convolution layer. The above two methods based on color image recognition can improve the target recognition rate more effectively than gray image recognition, but these methods ignore the cross-channel correlation of RGB. To overcome this shortcoming, a generalized discriminant model was proposed by Yang, converting three-color channels into one channel P, namely $P=x_{1}R+x_{2}G+x_{3}B$ [7]. In this method, the three-color channel is separated into three images, which are processed separately, and then weighted fusion. Although this method improves the level of face recognition to a certain extent, it is difficult to directly apply this method in practice because it is often difficult to obtain the optimized fusion basis $x_{1},x_{2},x_{3}$. In order to avoid solving the problem of fusion basis, Li proposed an integration method based on quaternion principal component analysis (QPCA) to solve the above problems [8] by importing quaternion to process three channels of color image at the same time, that is to say, a quaternion is used to represent the color image. Moreover, the quaternion complex representation is used to transform it from quaternion domain to complex domain; the quaternion matrix of color image in complex domain can be established, which greatly improves the recognition accuracy. However, dimensionality expansion increases the computational complexity, which affects the efficiency of recognition in complex scenarios and reduces the interpretability of principal components to recognition results.

Aiming at a sparse principal component of a color image, Lin proposed a quaternion sparse principal component analysis method based on $L_{1}$ norm for sparse optimization of quaternion principal components [9]. However, the principal components obtained are not sparse enough. When a face image has a large area occlusion, it is difficult to achieve ideal recognition accuracy and computational complexity. Moreover, in the practical application of face image recognition, occlusion is a relatively common type which is especially affected by COVID-19; mask occlusion is inevitable in various face recognition scenes. It is necessary to improve the algorithm recognition accuracy under occlusion.

Therefore, a color occlusion face recognition method based on the quaternion non-convex sparse constraint mechanism is proposed in this paper, and the quaternion non-convex sparse principal component analysis network model (QNSPCANet) is established. On the basis of the PCANet model structure, the quaternion representation method was used to construct the quaternion sample matrix of a color image. Then, $L_{p}$ non-convex regularization was used as the constraint term in the convolution kernel sparse optimization problem. $L_{p}$ regularization has a good feature extraction effect for face images with large area occlusion due to its strong sparsity [10]. Secondly, as the non-convex and non-smooth properties of the objective function built based on $L_{p}$ regularization, the coordinate descent method was used to solve the sparse principal components, and the fixed point iterative method was used to obtain the optimal numerical solution of the variables. Finally, experiments were performed on Georgia Tech, Color FERET, AR, and LFW-A face datasets. 

## 2. Main Research Work

**Figure 1:**
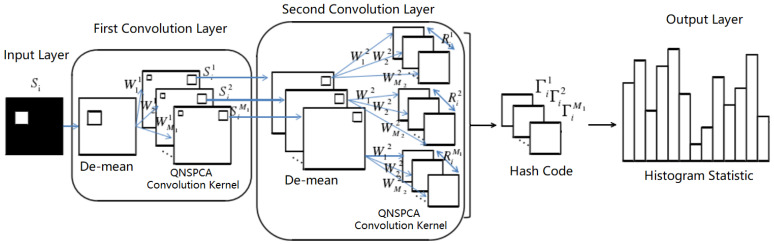
Basic framework of two-order QNSPCANet.

In order to overcome the problem that the convolution kernel of quaternion sparse principal component analysis is not sparse enough, the quaternion non-convex sparse principal component analysis network (QNSPCANet) based on $L_{p}$ regularization is proposed in this section: the establishment of the quaternion non-convex sparse PCANet model with $L_{p}$ regularization constraints, the quaternion sparse vector solution based on coordinate descent method, and the variable solution based on fixed point iteration method are proposed in order to obtain a more sparse convolution kernel and further improve the recognition performance. The basic framework of a two-order QNSPCANet is shown in Figure 1.

Since $L_{1/2}$ regularization has the ability of being the most sparse in nature among $L_{p}\left(0<p<1\right)$ regularization [10], $L_{1/2}$ regularization represents $L_{p}$ regularization to verify the performance of network model in actual simulation experiments.

### 2.1. The Establishment of QNSPCANet Model

#### 2.1.1. Quaternion Representation of Color Images

${\left\{S_{t}\right\}}_{t=1}^{N}$ denotes a set of training samples, and the size of each image is m×n. The red, green, and blue channel matrices of each color image $S_{t}$ are extracted and denoted as $R_{t},G_{t},B_{t}\in R^{m\times n}$, and the mean matrices $\langle R\rangle ,\langle G\rangle ,\langle B\rangle \in R^{m\times n}$ of all color images are respectively calculated, and ${\tilde{R}}_{t},{\tilde{G}}_{t},{\tilde{B}}_{t}\in R^{m\times n}$ are obtained after average processing of each image. $C_{t}$ can be regarded as the pixel matrix of the background color of the image, and it is the zero matrix when the background of the image is white. $Q_{t}\in H^{m\times n}$ is the quaternion matrix of each image, i.e.,:

(1) $$Q_{t}=C_{t}+{\tilde{R}}_{t}\cdot i+{\tilde{G}}_{t}\cdot j+{\tilde{B}}_{t}\cdot k$$

where i,j,k represent the three axes of the imaginary part of quaternion.

Then, the complex representation of quaternion is introduced, and the quaternion matrix is reconstructed into a general complex matrix. Let:

(2) $$\begin{array}{l} Q_{ta}=C_{t}+{\tilde{R}}_{t}\cdot i\\ Q_{tb}={\tilde{G}}_{t}+{\tilde{B}}_{t}\cdot i \end{array}$$

And the Equation (1) can be equivalently converted to:

(3) $$Q_{t}=Q_{ta}+Q_{tb}\cdot j$$

The complex representation of quaternion matrix is obtained by reconstruction.

(4) $$\chi_{t}=\left[\begin{array}{cc} Q_{ta} & Q_{tb}\\ -\bar{Q_{ta}} & \bar{Q_{tb}} \end{array}\right]$$

where $\chi_{t}\in C^{2m\times 2n}$, $-\bar{Q_{ta}}=-C_{t}+{\tilde{R}}_{t}\cdot i$, and $\bar{Q_{tb}}={\tilde{G}}_{t}-{\tilde{B}}_{t}\cdot i$.

Annotation 2.1: By reconstructing the color image input samples into the complex representation of quaternion, it can not only associate the color information of RGB three channels simultaneously, but also transform it from quaternion field to complex number field, which is convenient for the subsequent convolution layer operation. However, at the same time, the dimension of the input matrix is doubled and the computational complexity is increased.

#### 2.1.2. Quaternion Non-Convex Sparse Principal Component Analysis Convolution Kernel

It can be concluded from the previous section that the original input of the convolutional network is ${\left\{\chi_{t}\right\}}_{t=1}^{N}\in C^{2m\times 2n}$. Then, the sliding window of size $k_{1}\times k_{2}$ is used for block sampling of the t-th color image, and 2m×2n sample blocks can be obtained, where each sample block is a quaternion complex representation matrix. The t-th color image matrix $q_{t}=[q_{t,1},q_{t,2},\cdots ,q_{t,4mn}]\in C^{k_{1}k_{2}\times 4mn}$ is obtained by means of all sampling blocks and series processing. The same operation can be performed for each sample in ${\left\{\chi_{t}\right\}}_{t=1}^{N}$ to obtain the processed input sample matrix:

(5) $$X=\left[q_{1},q_{2},\cdots ,q_{N}\right]\in C^{k_{1}k_{2}\times 4Nmn}$$

Next, the quaternion sparse convolution kernel is calculated. Firstly, the covariance matrix of sample matrix X needs to be calculated. The calculation formula is as follows:

(6) $$\Phi =XX^{H}$$

where $X^{H}$ is the corresponding conjugate transpose of X. Then, Φ undergoes Eigendecomposition, and the Eigenvectors corresponding to the first $M_{1}$ largest Eigenvalues are retained as initial values, that is, $A=B=[\beta_{1},\beta_{2}\cdots ,\beta_{M_{1}}]\in C^{k_{1}k_{2}\times M_{1}}$, where $M_{1}\leq k_{1}\times k_{2}$. The quaternion non-convex sparse optimization problem is established as follows:

(7) $$\begin{array}{l} \left(\hat{A},\hat{B}\right)=\underset{A,B}{\arg}\min\left({\left\|X-AB^{H}X\right\|}_{F}^{2}+\lambda_{2}\sum_{j=1}^{M_{1}}{\left\|\beta_{j}\right\|}_{2}^{2}+\sum_{j=1}^{M_{1}}\lambda_{p,j}{\left\|\beta_{j}\right\|}_{p}^{p}\right)\\ s.t.A^{H}A=I_{M_{1}} \end{array}$$

where $\sum_{j=1}^{M_{1}}\lambda_{p,j}{\left\|\beta_{j}\right\|}_{p}^{p}$ represents the $L_{p}$ regularization constraint term, the sparsity of load $\beta_{j}$ is controlled by $\lambda_{p,j}$, $L_{p}$ norm is defined as ${\left\|x\right\|}_{p}=(\sum_{i}{\left|x_{i}\right|}^{p}{)}^{\frac{1}{p}}$, and $\lambda_{2}\sum_{j=1}^{M_{1}}{\left\|\beta_{j}\right\|}_{2}^{2}$ is to avoid the over-fitting problem. The quaternion sparse vector basis obtained after optimization solution is $V_{S}^{1}=[v_{s1}^{1},v_{s2}^{1}\cdots ,v_{sM_{1}}^{1}]\in C^{k_{1}k_{2}\times M_{1}}$, and, $v_{sj}^{1}=\beta_{j}/\left\|\beta_{j}\right\|\in C^{k_{1}k_{2}}$.

Therefore, the corresponding single QNSPCA convolution kernel is represented as follows:

(8) $$W_{l_{1}}^{1}=matrics_{k_{1},k_{2}}\left(v_{sl_{1}}^{1}\right)\in C^{k_{1}\times k_{2}}$$

where $l_{1}=1,2,\cdots ,M_{1}$, $matrics_{k_{1},k_{2}}\left(•\right)$ maps the vector to a matrix, and $v_{sl_{1}}^{1}$ represents the $l_{1}$-th principal component vector in the first layer quaternion sparse vector matrix.

#### 2.1.3. Two-Order Convolution Layer

The quaternion sparse convolution kernel calculated in the previous section is used to perform convolution operation with the sample image, then the output after the first convolution is:

(9) $$F_{t}^{l_{1}}=\chi_{t}*W_{l_{1}}^{1}$$

where $l_{1}=1,2,\cdots ,M_{1},t=1,2,\cdots ,N$. After a convolution, a color image $\chi_{t}$ can obtain $M_{1}$ corresponding Eigenmatrices, which serve as the input of the next layer.

The second convolution operation has the same principle as the first convolution, but the image size is reduced after convolution. Therefore, the edge of the matrix output of the previous layer needs to be zeroed before the second convolution, and the final output of the first convolution is ${\left\{F_{t}^{l_{1}}\right\}}_{l_{1}=1}^{M_{1}}\in C^{2m\times 2n}$. Then each Eigenmatrix $F_{t}^{l_{1}}$ is sampled by $k_{1}\times k_{2}$ sliding window, and 2m×2n sampling blocks can be obtained. The $l_{1}$-th sampling Eigenmatrix $z_{t}^{l_{1}}=[z_{t}^{l_{1},1},z_{t}^{l_{1},2},\cdots ,z_{t}^{l_{1},4mn}]\in C^{k_{1}k_{2}\times 4mn}$ is obtained by means removal and series connection of all sampling blocks. Then the same operation can be performed for each matrix in ${\left\{F_{t}^{l_{1}}\right\}}_{l_{1}=1}^{M_{1}}$, and the second layer input matrix $Z^{t}=\left[z_{t}^{1},z_{t}^{2},\cdots ,z_{t}^{M_{1}}\right]\in C^{k_{1}k_{2}\times 4M_{1}mn}$ corresponding to the t-th color image sample can be obtained. Finally, the sample matrix of the second convolution input of N image samples can be obtained.

(10) $$Z=\left[Z^{1},Z^{2},\cdots ,Z^{M_{1}}\right]\in C^{k_{1}k_{2}\times 4M_{1}Nmn}$$

Similarly, the quaternion sparse convolution kernel of the second convolution can also be calculated through Equations (6) and (7). The second QSPCA convolution kernel is expressed as follows:

(11) $$W_{l_{2}}^{2}=matrics_{k_{1},k_{2}}\left(v_{sl_{2}}^{2}\right)\in C^{k_{1}\times k_{2}}$$

where $l_{2}=1,2,\cdots ,M_{2}$,$v_{sl_{2}}^{2}$ represents the $l_{2}$-th principal component vector in the second quaternion sparse vector matrix.

For $M_{1}$ feature matrices output after the first convolution, each feature matrix $F_{t}^{l_{1}}$ corresponds to $M_{2}$ feature matrices after the second convolution

(12) $$R_{t}^{l_{1}}={\left\{F_{t}^{l_{1}}*W_{l_{2}}^{2}\right\}}_{l_{2}=1}^{M_{2}}$$

Therefore, each input sample $\chi_{t}$ can obtain $M_{1}\times M_{2}$ feature matrices after feature extraction of QSPCANet two-layer convolution. 

#### 2.1.4. Pooling and Feature Output

The quaternion sparse feature matrix obtained from the second-order convolution layer can be used as the feature output of the sample only after the pooling operation. Therefore, each feature matrix of the convolution output is input to the binarization function first, and then the binarization feature matrix is encoded. Each output feature matrix is obtained by convolving the input sample with different quaternion sparse convolution kernels. The larger the Eigenvalue of the convolution kernel is, the greater its contribution is. Therefore, the corresponding output feature matrix should also be given greater weight, and the weighted feature matrix can be obtained as follows:

(13) $$\Gamma_{t}^{l_{1}}=\sum_{l_{2}=1}^{M_{2}}2^{l_{2}-1}H\left(F_{t}^{l_{1}}*W_{l_{2}}^{2}\right)$$

where H(⋅) represents the given binarization function, that is, it is set to 0 when the modulus of the element is less than the given threshold, and 1 otherwise. After the above binarization hash coding, the $M_{1}\times M_{2}$ Eigenmatrices obtained by the second convolution become $M_{1}$ Eigenmatrices, and the pixel values in each Eigengraph are integers with a range of $\left[{0,\;2}^{M_{2}}-1\right]$.

Finally, we form the area histogram statistics for $M_{1}$ matrices corresponding to color image $\chi_{t}$. Each matrix is divided into C blocks, and then the statistical interval is set as $2^{M_{2}}$. The information in each histogram block is counted and connected in series to obtain the histogram feature $T_{t}^{l_{1}}\in R^{2^{M_{2}}C}$ of each feature matrix $\Gamma_{t}^{l_{1}}$. Finally, the histogram feature vector of each input sample is output as:

(14) $$f_{t}={\left[T_{t}^{1},T_{t}^{2},\cdots ,T_{t}^{M_{1}}\right]}^{T}\in R^{2^{M_{2}}CM_{1}}$$

The sample can output the final quaternion sparse feature matrix after feature extraction by QSPCANet. Then, in order to be consistent with other PCANet methods, the SVM classifier is also used to realize color face recognition. The advantage of SVM is that it can effectively solve the problems of a small sample, nonlinear and high-dimensional regression, and classification. Compared with the complexity of the problem, SVM requires a relatively small number of samples; a case where sample data are linearly indivisible can be solved by kernel function and relaxation variable. High dimension means that the sample dimension is very high, because the classifier generated by SVM is very simple, and the sample information only uses a support vector. At the same time, because SVM is only determined by support vector and has its own $L_{2}$ regularization, it can effectively prevent the over-fitting problem. 

Annotation 2.2: the QNSPCANet method described in this section is a new network model proposed in this chapter, especially the $L_{p}$ regularization of strong sparsity. Moreover, the computational complexity of QNSPCANet feature extraction is $4mnM_{1}\left(M_{2}+1\right)k_{1}k_{2}$, and the convolution storage space is $8k_{1}k_{2}\left(M_{1}+M_{2}\right)$ bytes. Compared with other methods, QNSPCANet has the following advantages:

(1) QNSPCANet uses $L_{p}$ regularization to compute sparse convolution kernels, which has higher sparse efficiency and can reduce computational complexity compared with general $L_{1}$ regularization;
(2) Sparse regularization is beneficial to identify important variables related to outliers, while the principal component convolution check outliers calculated by non-convex regularization of strong sparsity have better robustness and improve model recognition performance;
(3) For the image with occlusion, the sparse principal component convolution kernel can reduce the influence of outliers in the occlusion area and further improve the recognition accuracy.

However, the QNSPCANet model established in this section has non-convex and non-smooth problems in sparse optimization, which makes it difficult to solve. Although the establishment of an alternate solution model can effectively overcome the difficulty of solving two variables simultaneously and reduce the complexity to a certain extent, it still cannot overcome the essential difficulty brought by the introduction of $L_{p}$ norm. Therefore, we discuss the solution method for $L_{p}$ non-convex optimization problems in the next section.

### 2.2. Lp Non-Convex Sparse Optimization Method for Model Parameters

In order to overcome the difficulty in solving the parameters of QNSPCA convolution kernel, the variables of Equation (7) are first divided into two coordinate blocks, A and B, and one coordinate block is fixed to solve the sub-problems of the other coordinate block, and the sub-problems of the two variables are solved in turn until the termination condition is met [11]. In the algorithm, the initial value of A and B are the first k principal components obtained from QPCA, that is, $A=B=[v_{1},v_{2}\cdots ,v_{k}]$.

Fixing A to solve problem (7) is equivalent to solving:

(15) $$\hat{B}=\underset{B}{\arg}\min\left({\left\|XA-XB\right\|}_{F}^{2}+\lambda_{2}\sum_{j=1}^{k}{\left\|\beta_{j}\right\|}_{2}^{2}+\sum_{j=1}^{k}\lambda_{1,j}{\left\|\beta_{j}\right\|}_{p}^{p}\right)$$

Y=XA, and $y_{j}=Xv_{j},j=1,\ldots ,k$ is initialized, then solving Equation (15) is equivalent to solving k independent optimization problems:

(16) $${\hat{\beta }}_{j}=\underset{\beta_{j}}{\arg}\min\left({\left\|y_{j}-X\beta_{j}\right\|}_{F}^{2}+\lambda_{2}{\left\|\beta_{j}\right\|}_{2}^{2}+\lambda_{1,j}{\left\|\beta_{j}\right\|}_{p}^{p}\right)$$

Based on the obtained B, the singular value decomposition of $XX^{H}B$ is calculated:

(17) $$XX^{H}B=UDV^{T}$$

And the A is updated:

(18) $$\hat{A}=UV^{T}$$

B and A can continue to be solved alternately until the convergence.

Annotation 2.3: the establishment of the alternate solution model can effectively overcome the difficulty of solving two variables at the same time and reduce the complexity to a certain extent, but it still cannot overcome the essential difficulty caused by the introduction of the $L_{p}$ norm. Therefore, we introduce the coordinate descent method and fixed point iterative method in the next section to overcome this difficulty. 

#### 2.2.1. Coordinate Descent

Coordinate descent (CD) [12] is a simple but efficient optimization algorithm. It does not calculate gradient, but minimizes the objective function along the direction of each coordinate axis; that is, only one coordinate direction is found at this point and the remaining coordinate directions are kept unchanged. Then we can iterate over each coordinate direction until we obtain the local minimum.

If there is an objective function $f\left(x_{1},x_{2},\cdots ,x_{n}\right)$, and it needs to solve its minimum point, $x=[x_{1},x_{2},\cdots ,x_{n}]$, x is initialized, it is called $x^{0}$, and then the cycle is started, the iteration process of the i(i=1,2,⋯,n)-th dimension in the t-th cycle is as follows:

(19) $$x_{i}^{\left(t\right)}=\underset{x_{i}}{\arg\min}f\left(x_{1}{}^{\left(t\right)},x_{2}^{\left(t\right)},\cdots ,x_{i-1}^{\left(t\right)},x_{i},x_{i+1}^{\left(t-1\right)},\cdots ,x_{n}^{\left(t-1\right)}\right)$$

It is equivalent to solving $x_{i}$ only as a variable in each iteration, while the remaining n−1 dimensions are regarded as constants and keep the current value unchanged. Then, $f\left(x_{1},x_{2},\cdots ,x_{n}\right)$ is minimized to obtain the new value of $x_{i}$ and it is substituted into the next iteration as a constant.

If the relative changes in $x^{\left(t\right)}$ and $x^{\left(t-1\right)}$ in each dimension are less than the specified threshold, the $x^{\left(t\right)}$ is the final result. Otherwise, the cycle continues for the t+1-th time until it is less than the change threshold or reaches the maximum number of cycles, and finally reaches the local minimum point.

For k principal components, these are k independent problems, so we only need to provide the coordinate descent solution algorithm for the first optimization problem, and so on for the other k−1 problems. Without loss of generality, the subscripts of $\beta_{j}$ and $y_{j}$ are omitted, i.e., $\beta \in C^{k_{1}k_{2}\times 1},y\in C^{4Nmn\times 1}$. Equation (16) can be simplified as

(20) $$\hat{\beta }=\underset{\beta }{\arg}\min\left({\left\|X^{H}\beta -y\right\|}_{2}^{2}+\lambda_{2}{\left\|\beta \right\|}_{2}^{2}+\lambda_{p}{\left\|\beta \right\|}_{p}^{p}\right)$$

The coordinate descent method is applied to Equation (20). In the process of each cycle iteration, only one target variable is minimized and the values of other variables are fixed, which is equivalent to solving the unitary optimization problem. Therefore, for the i-th component $\beta_{i},i=1,2,\cdots ,k_{1}k_{2}$ of β, problem (20) is solved, the current value of $\beta_{s}\left(s\neq i\right)$ is unchanged, and only $\beta_{i}$ is optimized in each iteration, which is equivalent to solving:

(21) $${\hat{\beta }}_{i}=\underset{\beta_{i}}{\arg}\min\left(\left(\lambda_{2}+\sum_{j=1}^{4Nmn}x_{ij}^{2}\right)\beta_{i}^{2}-\left(\sum_{j=1}^{4Nmn}x_{ij}r_{j}^{\left(i\right)}\right)\beta_{i}+\lambda_{p}{\left|\beta_{i}\right|}^{p}\right)$$

where $r_{j}^{\left(i\right)}=y_{j}-\sum_{s\neq i}^{k_{1}k_{2}}x_{sj}\beta_{s},j=1,2,\cdots ,4Nmn$, that is, the residual of $y_{j}$ is fitted only with other fixed variables.

Annotation 2.4: In reference [11], this iterative updating method is called trivial updating, and the computational complexity of this method is O(mn). The coordinate descent method adopted in this paper has low complexity, and the number of cycles selected by the algorithm termination condition is less than the maximum number of cycles, or the update rate of the objective function value is less than the given threshold. However, in the process of solving a single component, the introduction of $L_{p}$ regularization is still unavoidable.

#### 2.2.2. Fixed Point Iterative Method

In order to overcome the univariate solving problem caused by $L_{p}$ regularization, the fixed point iterative method is used to optimize the numerical solution in this section.

**Theorem** **1.** *Given a function* $f\left(x_{1},\cdots ,x_{i},\cdots ,x_{n}\right)$*, suppose*$x_{i}$*exists such that*$x_{i}=g\left(x_{i}\right)$*, then the point*$x_{i}$*is a fixed point of*$f\left(x_{1},\cdots ,x_{i},\cdots ,x_{n}\right)$.

For Equation (21), $f\left(\beta_{1},\cdots ,\beta_{i},\cdots ,\beta_{n}\right)=b\beta_{i}{}^{2}-a\beta_{i}+\lambda_{p}{\left|\beta_{i}\right|}^{p}$, the first derivative equation of it can be expressed as follows:

(22) $$\begin{array}{l} {\left(b\beta_{i}{}^{2}-a\beta_{i}+\lambda_{p}{\left|\beta_{i}\right|}^{p}\right)}^{\prime }=0\;\\ =>\left\{ \begin{array}{l} 2b\beta_{i}-a+p\lambda_{p}\beta_{i}{}^{p-1}=0\;\;\;\beta_{i}>0\\ 2b\beta_{i}-a-p\lambda_{p}{\left(-\beta_{i}\right)}^{p-1}=0\;\beta_{i}<0 \end{array}\right. \end{array}$$

Since the function is non-convex after $L_{p}$ regularization is added, the variable optimization solution cannot be obtained directly from the first derivative equation. Therefore, the fixed point iteration method is used to transform the first-order derivation problem into the fixed point iteration problem $\beta_{i}=g\left(\beta_{i}\right)$, and the equivalent deformation of Equation (22) can be obtained:

(23) $$\beta_{i}=\left\{ \begin{array}{l} \frac{a}{2b}-\frac{p\lambda_{p}}{2b}\beta_{i}{}^{p-1}\;\;\beta_{i}>0\\ \frac{a}{2b}+\frac{p\lambda_{p}}{2b}{\left(-\beta_{i}\right)}^{p-1}\;\beta_{i}<0 \end{array}\right.$$

Therefore:

(24) $$g\left(\beta_{i}\right)=\left\{ \begin{array}{l} \frac{a}{2b}-\frac{p\lambda_{p}}{2b}\beta_{i}{}^{p-1}\;\;\beta_{i}>0\\ \frac{a}{2b}+\frac{p\lambda_{p}}{2b}{\left(-\beta_{i}\right)}^{p-1}\;\beta_{i}<0 \end{array}\right.$$

where $b=\lambda_{2}+\sum_{j=1}^{4Nmn}x_{ij}^{2}\;$, $a=\sum_{j=1}^{4Nmn}x_{ij}r_{j}^{\left(i\right)}$. The initial value of the input variable is taken as the initial iteration value, and then the numerical solution is iterated. Therefore, the numerical solution obtained iteratively by using Equation (23) is the optimal solution ${\hat{\beta }}_{i}$ of $\beta_{i}$ under the current cycle.

Annotation 2.5: the fixed point iterative method is an important method for solving nonlinear equations [13]. After transformation into fixed point equation, approximate solution of the equation can be obtained through iteration, which is not limited by nonlinear and non-convex conditions. Therefore, this section adopts fixed point iteration method to solve variables. 

## 3. Algorithm Simulation Experiments

**Figure 2:**
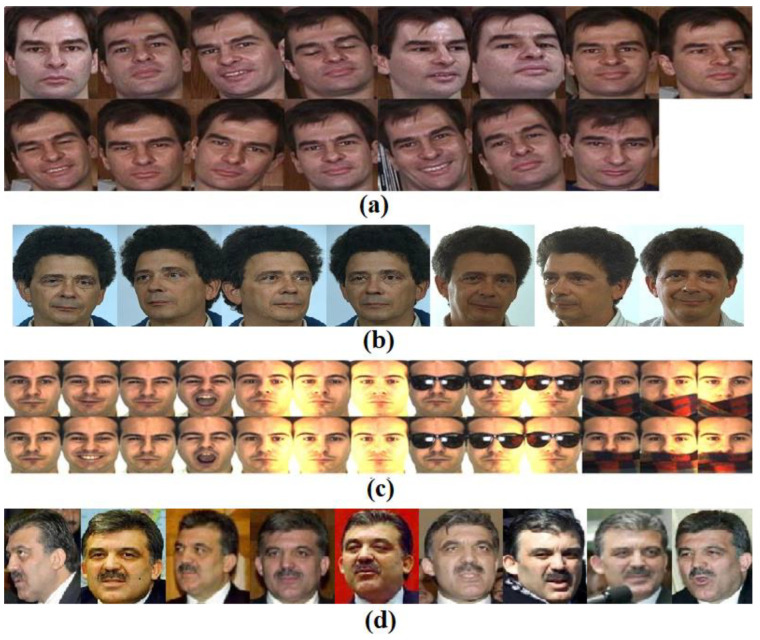
Sample for color face image (**a**) Georgia Tech; (**b**) Color FERET; (**c**) AR; (**d**) and LFW-A.

In this section, four color face datasets including Georgia Tech [14], Color FERET [15], AR [16], and LFW-A [17] were selected to conduct algorithm simulation experiments. Color face image samples of four datasets are shown in Figure 2.

The Georgia Tech dataset consists of 750 color images, 15 images for each of 50 photographers, and is widely used in face recognition. The face images were taken in the Georgia Tech Lab, and most of the images included changes in light, expression, and posture.

Color FERET is a Color version of the gray face dataset FERET, with 11,338 color face images from 994 people. Since the number of images varies from person to person, 200 photographers were selected with seven images each, and the 1400 images were recorded as the Color FERET subset. Seven images of each person were selected with changes in lighting, expression, and posture.

AR is a face dataset composed of 3120 color face images, 26 images per 120 photographers. Face images were taken from the front, so the pose changes are relatively few. In addition to the illumination and expression changes, the influence of occlusion factors is also considered.

LFW-A is a version of LFW face dataset after face alignment processing, which includes 13,233 color images from 5749 photographers. As the images come from the network, it is suitable for face recognition research in natural scenes. The face images in the dataset include a variety of factors such as illumination, age, posture, expression, and occlusion, so the dataset is very challenging. In order to evenly distribute samples, photographers with more than nine images were selected and A subset of LFW-A was constructed. 

The images of each person in each database were randomly divided into the training set and test set in accordance with 2:1. Since the distribution of training samples and test samples in the experiment was random, the experiment was repeated for ten times and the average value was taken. In order to test more effectively in the actual experiment, the size of all images was uniformly set as 32×32.

Meanwhile, based on the experimental situation in this paper, the SVM classifier chose the LIBLINEAR library based on linear kernel function. The linear kernel is suitable for cases where the number of samples is much smaller than the number of features (no need to map to higher dimensions), or where both the number of samples and the number of features are large (mainly considering the training speed).

In addition, the hardware of this experiment is Intel(R) Core (TM) i5-8265U CPU@1.80 GHz, NVIDIA GTX 1060, and the software is Matlab 2016b and Anaconda3.

### 3.1. Comparison of Algorithm Performance under Different Occlusion Conditions

Firstly, the QNSPCANet proposed in this paper and the three algorithms, PCANet, QPCANet, and QSPCANet, were compared under different occlusion conditions. Different occlusion conditions mainly include occlusion contained in the dataset, self-added pure color occlusion, and salt-and-pepper noise occlusion. At the same time, Color FERET, AR, and LFW-A datasets also compared the recognition accuracy of other latest occlusion algorithms under the same dataset. The Georgia Tech dataset was only compared with similar structure algorithms due to its few applications.

Since there are no large-area occlusion elements in the face images of Georgia Tech, Color FERET, and LFW-A datasets, the experiments of these three datasets can be divided into three groups: (1) when the first group is without occlusion, the images of the training set and test set are randomly selected in 2:1; (2) in the second group, a blue block with 20% pixel area was added to the randomly selected face images in the test set; (3) and the third group is the condition of salt-and-pepper noise occlusion. Salt-and-pepper noise blocks are added to randomly selected face images in the test set.

**Figure 3:**
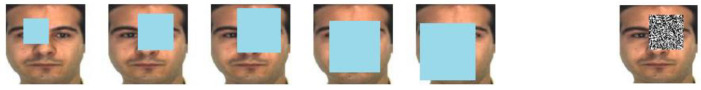
Color face sample of AR dataset and self-added occlusion processing example.

For AR dataset, each person should have 26 face images, among which, eight have no occlusion factor, six have illumination change, six have sunglasses occlusion, and six have scarf occlusion. The experiment can be divided into five groups: (1) in the first group, under the condition of no occlusion, nine images were randomly selected from 14 images with no occlusion containing light changes to form the training set, and the remaining five images were formed into the test set; (2) in the second group, 14 face images without occlusion were included in the training set, and six face images with sunglasses occlusion were included in the test set; (3) the third group was scarf occlusion, 14 images without occlusion were used as the training set, and six images with scarf were used as the test set; (4) the fourth group was in the condition of self-added pure color occlusion. The training set consisted of nine randomly selected face images without occlusion, and then added blue occlusion blocks (about 20% occlusion area) to the remaining five images, which were used as test samples; (5) and the fifth group was the condition of salt-and-pepper noise occlusion. Nine images were randomly selected from 14 images without occlusion as the training set, and then the remaining five images were added with salt-and-pepper noise blocks as the test set. Color face samples of the AR dataset, self-added solid color occlusion of images, and different areas of salt-and-pepper noise occlusion are shown in Figure 3. 

The convolution order of each principal component analysis network is set as 2, the number of convolution kernels at each layer is $M_{1}=M_{2}=8$, the size of sampling matrix is set as the optimal size of each dataset, the size of histogram window is set as 7×7, and the corresponding overlap rate is set as 0.5. For the quaternion sparse optimization problem in the convolution layer, $\lambda_{2}=0.001$ is set, the appropriate sparse parameter $\lambda_{p},\lambda_{s}$ for each feature vector is selected, the update rate threshold is set as 1e−4, and the maximum cycle time is set as 1000. Finally, a trained SVM classifier is used for color face recognition based on the extracted features.

Table 1 shows the correct recognition rate of each algorithm in Georgia Tech dataset under different occlusion conditions.

It can be seen from Table 1 that the algorithm introducing $L_{p}$ non-convex regularization achieves a relatively high recognition rate under different occlusion conditions. When there is no occlusion, the recognition rate of the two algorithms under sparse constraint is close. In the case of 20% pure color occlusion area and salt-and-pepper noise occlusion, the recognition rate of QNSPCANet is the highest. Due to the few occlusion applications in Georgia Tech dataset, this part is only compared with the relevant PCANet method.

The correct recognition rate of each algorithm in Color FERET dataset under different occlusion conditions is shown in Table 2.

As can be seen from Table 2, in the Color FERET dataset, QNSPCANet achieves the highest recognition rate in the case of non-noise occlusion. In the case of 20% salt-and-pepper noise, the recognition rate is slightly lower than GMSRC, but the overall recognition performance is still superior. 

The correct recognition rate of each algorithm in AR dataset under different shielding conditions is shown in Table 3. The recognition rate of all algorithms in AR dataset is generally higher. QNSPCANet has the best recognition effect under all occlusion conditions, and the difference of recognition rate is larger when there is occlusion, indicating that $L_{p}$ regularization has a good suppression effect on both outliers and occlusion. 

The correct recognition rate of each algorithm in LFW-A dataset under different shielding conditions is shown in Table 4. It can be seen from the table that QNSPCANet, which introduced non-convex regularization, has a higher recognition rate than PCANet and QPCANet under different occlusion conditions. Compared with QSPCANet based on $L_{1}$ regularization, the recognition rate is close to that of QSPCANet without occlusion, while the recognition rate is greatly improved with occlusion. At the same time, compared with other existing methods in the table, the algorithm proposed in this paper can also achieve better recognition effect. Although there is still a certain gap between QNSPCANet and MobileFaceNet when there is no occlusion, QNSPCANet has better performance when there is occlusion and noise.

**Figure 4:**
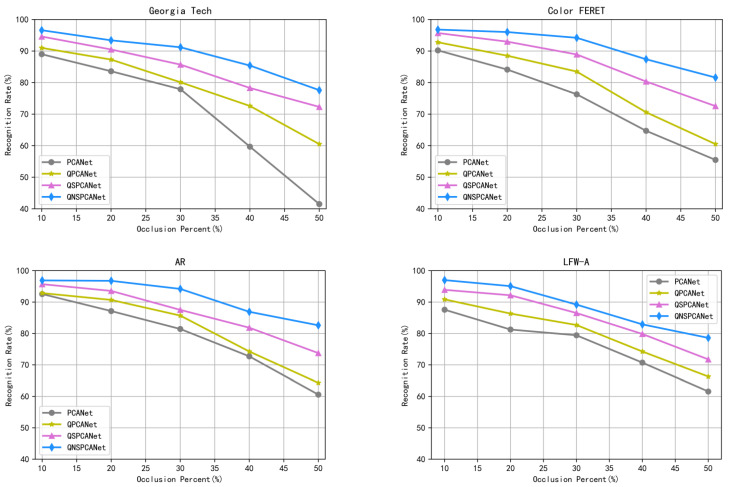
The recognition rate curves of each algorithm under different solid color occlusion areas.

Figure 4 shows the recognition rate curves of each algorithm under different solid color occlusion areas. As can be seen from the figure, with the increase in the occlusion area, the gap between the correct recognition ability of the four recognition algorithms gradually increases and QNSPCANet performs better under different occlusion areas.

Since no specific training time is provided in the literature of other occlusion methods, this paper only compares the training time of the four PCANet algorithms, as shown in Table 5.

As can be seen from Table 5, the overall training time of the four PCANet related algorithms is short. The training time of the QNSPCANet method proposed in this paper is increased, mainly because non-convex sparse optimization produces certain calculation consumption, but the recognition accuracy is improved, and the overall recognition performance is still superior. 

Based on the above comparative experimental results, it can be shown that in the case of no occlusion, the recognition rate of non-convex sparse convolution check is improved, but the effect is not obvious because the image contains fewer outliers at this time, which has a slight impact on model recognition results. In the case of occlusion, the strong sparse performance of non-convex regularization can effectively reduce the influence of occlusion outliers to improve the identification accuracy of the model.

### 3.2. Algorithm Sparsity Verification

Secondly, the sparsity of QNSPCANet proposed in this paper is verified. The validation experiment is mainly carried out on AR datasets and compared with $L_{1}$ regularization and SCAD regularization [25]. In order to test the sparsity of $L_{1/2}$ non-convex regularization ($L_{p}$ is the most representative of sparsity), this section adopts two sparsity measures: the proportion of non-zero elements in the sparse matrix and Hoyer′s sparsity measure. Hoyer’s sparsity measures the components of small values and is a more refined sparse measurement method than non-zero values, which is defined as:

(25) $$Hoyer\left(V_{S}^{i}\right)=\frac{\sqrt{M_{i}k_{1}k_{2}}-{\left\|V_{S}^{i}\right\|}_{1}/{\left\|V_{S}^{i}\right\|}_{F}}{\sqrt{M_{i}k_{1}k_{2}}-1}\in \left[0,1\right]$$

where $V_{S}^{i}$ represents the quaternion sparse vector matrix in the i-th convolution layer, and $M_{i}k_{1}k_{2}$ represents the number of elements of the matrix.

**Figure 5:**
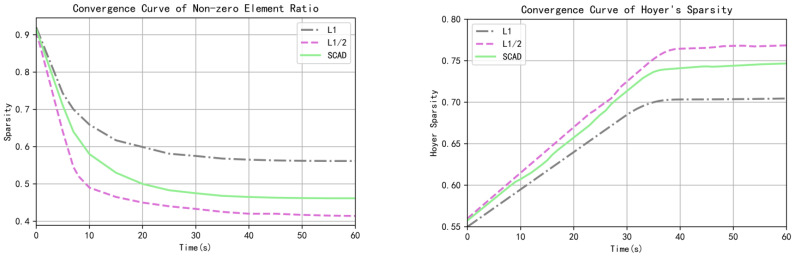
Convergence curve of sparsity of the first convolution layer sparse vector matrix.

The proportional convergence curve of non-zero elements of the sparse matrix and the convergence curve of Hoyer′s sparsity are shown in Figure 5.

As can be seen from Figure 5, the proportion of $L_{1}$ regularization non-zero elements is 0.56, while the Hoyer’s sparsity is about 0.70. The proportion of non-zero elements convergent in $L_{1/2}$ regularization is 0.41, and the Hoyer’s sparsity is about 0.77. The proportion of non-zero elements regularized by SCAD is 0.46, and the Hoyer’s sparsity is about 0.75. Compared with $L_{1}$ regularization, $L_{1/2}$ regularization decreased by 26.8% and SCAD regularization decreased by 17.9%. Compared with $L_{1}$ regularization, $L_{1/2}$ regularization improved Hoyer′s sparsity by 10.0%, while SCAD regularization improved by 7.1%. Therefore, $L_{1/2}$ regularization has stronger sparsity than SCAD and $L_{1}$ regularization. 

### 3.3. Algorithm Robustness Verification

In order to verify the robustness of the model under $L_{1/2}$ non-convex regularization, different samples were randomly selected to form the training set and test set under different salt-and-pepper noise occlusion areas. The input model was repeated ten times and the corresponding root mean square error (RMSE) was calculated. Root mean square error can effectively reflect the stability of the model for random sample changes, and its calculation results are shown in Table 6. 

**Figure 6:**
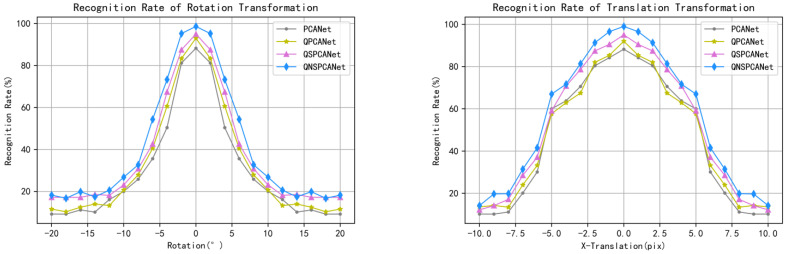
Four algorithms correctly identify rate curves under rotation transformation and translational transformation.

Then, a certain degree of rotation transformation and translation transformation were carried out on the samples of the test set to construct similar samples to test the stability of the model for transformation. For the image rotation transformation processing, the overall rotation angle increases within the range of $-20^{\circ }∼+20^{\circ }$; for translation transformation processing, −10∼+10 pixels of image can be translated based on the horizontal direction. Figure 6 shows the correct recognition rate curves of the four algorithms under different rotation and translational transformations. 

Combined with the results in Table 5 and Figure 6, it can be shown that QNSPCANet still has a low root mean square error in the case of noise. Meanwhile, QNSPCANet has good robustness to translation and rotation changes in samples. It can be seen that the proposed algorithm has higher stability, and the model is more robust.

## 4. Conclusions

In this paper, we propose a QNSPCANet model based on quaternion non-convex sparse constraint mechanism for color face image recognition with large area occlusion. $L_{p}$ regularization is introduced into PCANet as a constraint term in the convolution layer convolution kernel sparse optimization problem, and the non-convex optimization problem is innovatively converted into fixed point equation, avoiding the problem that the coordinate descent method cannot be directly derived in the single variable solution. Sparse regularization recognition of outliers can effectively solve the problems of face image occlusion and noise, and the strong sparsity of non-convex regularization can further improve the recognition performance of the model.

In order to verify the recognition performance of QNSPCANet model proposed in this paper, especially in the case of occlusion and noise, self-added occlusion and noise processing are performed on Georgia Tech, Color FERET, AR, and LFW-A Color face datasets and compared with PCANet, QPCANet, QSPCANet, and other latest occlusion algorithms in the same dataset. The experimental results show that the quaternion non-convex sparse principal component analysis network proposed in this chapter has a high recognition rate under different occlusion conditions. This paper also verifies the sparsity and robustness of $L_{p}$ regularization, and further proves the sparsity and robustness of $L_{p}$ regularization through experiments of sparsity, root mean square error, rotation, and translation transformation, etc. 

The PCANet framework based on this paper adopts a relatively simple number of network structure layers and lacks deeper feature extraction. If the deep features are acquired only by increasing the layers of the network model, the number of parameters and computational complexity will also be greatly increased. Therefore, it is necessary to build a new framework that is more advanced, simple and can extract deeper features. 

## Figures and Tables

**Table 1:** The correct recognition rate of each algorithm in Georgia Tech dataset under different occlusion conditions (%).

Algorithm	Normal	20% Block Occlusion	20% Noise Occlusion
PCANet	93.20	83.60	83.00
QPCANet	95.50	87.30	87.70
QSPCANet	97.50	92.50	91.40
QNSPCANet	97.70	95.40	94.40

**Table 2:** The correct recognition rate of each algorithm in Color FERET dataset under different occlusion conditions (%).

Algorithm	Normal	20% Block Occlusion	20% Noise Occlusion
FRAD [18]	96.59	94.80	94.33
GMSRC [19]	97.07	95.21	95.42
DDRC [20]	98.60	94.53	-
PCANet	93.75	84.13	84.42
QPCANet	96.44	88.52	88.11
QSPCANet	98.04	92.98	92.38
QNSPCANet	98.72	96.02	95.39

**Table 3:** The correct recognition rate of each algorithm in AR dataset under different occlusion conditions (%).

Algorithm	Normal	Sunglasses	Scarf	20% Block Occlusion	20% Noise Occlusion
Gabor-SRC [21]	96.72	95.83	95.26	92.41	92.89
VGG-Face [22]	98.05	97.21	93.40	89.47	91.55
Lightened-CNN [22]	98.79	98.14	98.56	96.01	-
PCANet	96.71	95.83	96.12	87.14	88.32
QPCANet	97.83	97.66	96.23	90.69	90.94
QSPCANet	99.41	98.50	98.74	93.56	92.71
QNSPCANet	99.62	99.25	99.31	96.77	95.80

**Table 4:** The correct recognition rate of each algorithm in LFW-A dataset under different occlusion conditions (%).

Algorithm	Normal	20% Block Occlusion	20% Noise Occlusion
ProCRC [23]	94.82	86.77	88.51
CRDDL [23]	95.20	89.56	90.13
MobileFaceNet [24]	98.20	90.53	95.08
PCANet	91.56	80.25	80.64
QPCANet	94.15	86.33	87.59
QSPCANet	97.10	92.17	93.36
QNSPCANet	97.35	95.08	95.21

**Table 5:** Algorithm training time comparison.

Algorithm	Georgia Tech	Color FERET	AR	LFW-A
PCANet	49.06 s	81.82 s	210.27 s	63.15 s
QPCANet	156.24 s	277.52 s	342.86 s	180.44 s
QSPCANet	194.36 s	325.58 s	481.39 s	237.89 s
QNSPCANet	226.57 s	369.14 s	510.44 s	268.51 s

**Table 6:** Root mean square error of five recognition algorithms under different salt-and-pepper noise occlusion area (%).

Algorithm	10%	20%	30%	40%	50%
PCANet	4.20	5.73	7.82	9.02	9.85
QPCANet	2.51	4.26	5.33	7.84	9.26
QSPCANet	0.94	1.12	1.58	1.93	2.29
QNSPCANet	0.79	0.98	1.24	1.57	2.01
